# Editorial: Ion Homeostasis in Plant Stress and Development

**DOI:** 10.3389/fpls.2020.618273

**Published:** 2020-11-20

**Authors:** José M. Mulet, Francisco Campos, Lynne Yenush

**Affiliations:** ^1^Instituto de Biología Molecular y Celular de Plantas, Universitat Politècnica de València-Consejo Superior de Investigaciones Científicas, Valencia, Spain; ^2^Departamento de Biología Molecular de Plantas, Instituto de Biotecnología, Universidad Nacional Autónoma de México, Cuernavaca, Mexico

**Keywords:** ion homeostasis, plant stress and development, drought stress, halotropism, chloride, plant hormones, calcium channels, purple acid phosphatases

Ion homeostasis is a dynamic process and a fundamental requirement for all organisms. Many different minerals are required for essential biochemical processes, but accumulation of these elements may be toxic. Thus, all living organisms have developed efficient systems to acquire and store these elements and maintain their cytosolic and organellar concentrations within a specific physiological range that allows for normal development. This requirement has determined the establishment of evolutionarily-conserved, robust molecular mechanisms to maintain these homeostatic concentrations and avoid toxicity, while at the same time, permitting dynamic responses to environmental changes. Some of these responses are conserved among different kingdoms, while others are specific for plants (Mulet et al., 2013). The presence of high ionic concentrations in soil, especially of sodium chloride, is a great conundrum for the plant's physiology, as cells must maintain potassium concentration high and sodium low inside the cytoplasm, usually against high sodium concentrations in the soil. Although saline habitats are common and diverse, halophytic plants comprise less of 2% of all gymnosperms (Flowers and Colmer, 2015). On the other hand, salt tolerant species have emerged in at least 100 different species of flowering plants (Santos et al., 2016). Some authors have suggested that salt tolerance may be a macroevolutionary self-destructive trait, as it is gained frequently, but also is lost easily by reversal or extinction (Bromham et al., 2020). This is probably due to the high amount of energy required to maintain the ion homeostasis under stress conditions, which hampers normal plant development and compromises the response to other environmental cues, the adaptation to new conditions or the competition with other species for the ecological niche.

Ion homeostasis determines pivotal functions for plant biology, such as the compensation of the negative charges of macromolecules, maintenance of electroneutrality, and the establishment of cell turgor and volume. Also, a proper ion potential in the internal media or in organelles is required for enzyme activity and other essential functions like protein synthesis. Ions are also essential components of biomolecules, such as chlorophyll or hemoglobin, and they play a key role at the whole plant level by contributing to vital processes, such as stomatal aperture which controls transpirational water loss, plant desiccation and cell elongation. In addition to direct effects of ions on plant cell physiological function and homeostasis, a proper membrane potential (inside negative) is maintained through the maintenance of specific cation and anion gradients across the cell membrane.

## Ion Homeostasis under Drought Stress

Drought is the main limiting factor for agricultural yield worldwide. The problem is increasing due to the rising water demand to feed a growing population and the effects of climate change, especially in drought-prone arid and semi-arid areas (Mahajan and Tuteja, 2005). In response to drought, plants activate a defense mechanism devoted to the accumulation of water and potassium. The mini-review presented by Nieves-Cordones et al. on this issue, summarizes the role of potassium and chloride in water deficit resistance in the different plant organs. High activity of K^+^ and Cl^−^ uptake systems and a large root system are desirable traits. In the leaf, K^+^ and Cl^−^ allow for an efficient osmotic adjustment of leaf cells, which is a key process to retain water within cells. Efficient stomatal closure prevents excessive water loss and is achieved by K^+^ and Cl^−^ release from guard cells. In fact, it has been postulated that proteins regulating potassium uptake channels in the guard cells may be targets for biotechnological strategies against drought (Locascio et al., 2019). The study of chloride homeostasis has often been neglected due to the importance of potassium, which is the major ion in the cytoplasm, but Cl^−^ has a specific beneficial effect in leaf cells by giving rise to larger cells (with higher water storage capacity), lower stomatal conductance and higher mesophyll conductance to CO_2_ (Dreyer and Uozumi, 2011; Colmenero-Flores et al., 2019). Therefore, water use efficiency is increased under proper Cl^−^ nutrition.

## Hormonal Aspects of Ion Homeostasis

The mini-review by Nieves-Cordones et al. also highlights the role of abscisic acid (ABA) for maintaining ion homeostasis under drought stress. ABA triggers K^+^ and Cl^−^ retention in roots, induces changes in root system architecture by enhancing lateral root growth and inhibiting primary root growth, and is induces stomatal closure. However, less is known about the interplay between ABA and other hormones, specifically auxin, during stress. This important aspect is the focus of the other mini-review included in this topic, written by Szepesi. Halotropism is a recently discovered movement phenomena that allows plants to avoid high salt by root bending, or in halophytic plants, remodel their root system architecture to find the optimal sodium concentration in the soil (Galvan-Ampudia et al., 2013). Auxin and ABA are essential for this process. Ethylene may also participate promoting growth under adverse conditions. It has also been shown that application of strigolactone hormones causes changes in root system architecture, therefore suggesting a role in halotropism (Ruyter-Spira et al., 2011).

## Calcium and Phosphate Homeostasis

At the center of the interplay between ion homeostasis and hormonal signaling, we find calcium homeostasis. Calcium acts as an intracellular second messenger during hormonal responses. For instance, during halotropism, elevated Na^+^ triggers the activation of a phospholipid signaling pathway by increasing Ca^2+^ levels in the cytoplasm (Korver et al., 2020). In the paper by Zhang et al., a new player in calcium homeostasis is unveiled. In this article, the authors use electrophysiological studies to show that the cyclic nucleotide-gated channel CNGC12 is a calcium transporter regulated by calmodulin.

Calcium homeostasis also affects phosphate homeostasis. Phosphate is an essential nutrient and, in many environments, it is the most limiting element. Plants have developed a complex network for the uptake and internal transport of phosphate, which is required for the synthesis of pivotal molecules such as DNA, RNA, phospholipids, and is involved in signaling networks regulated by phosphorylation/dephosphorylation. Calcium, like phosphate, is essential, but calcium has a high affinity for phosphate, producing calcium phosphate. Therefore, phosphate and calcium levels must be tightly regulated to avoid the formation of these insoluble salts. The report by Farhadi et al., gives a new insight into the phosphate homeostasis, as describes a new compensatory mechanism between two purple acid phosphatases is described, pointing out the importance of studying these proteins as part of a network and not as individual phosphatases.

## Outlook

We have gained a lot of knowledge regarding the function and regulation of proteins that participate in ion homeostasis and their role in salt and drought stress. Future investigation must address important questions related to the relationship between ion homeostasis and growth, development and stress response pathways. In addition, it is important to expand our understanding of the molecular mechanisms through which hormones controlling growth and development impact the regulation of ion homeostasis. For example, studies on the role of auxins or strigolactones in ion homeostasis have frequently been neglected due to the major and well-characterized role of ABA in ion homeostasis during stress responses. This Research Topic has contributed to these aspects by offering two minireviews which discuss the role of hormones in the regulation of ion homeostasis during growth and stress, and with two research papers that describe the co-regulation of proteins involved in Ca^2+^ and phosphate homeostasis. These studies help to shed light on the complex regulatory networks underlying ion homeostasis. We hope future investigation will help to solve the many questions that remain unanswered in this field, which is increasingly important for the successful development of the biotechnological approaches needed for effective agriculture in the face of climate change.

## Author Contributions

JMM wrote the first draft, based on it LY wrote the second draft, and then FC wrote the final version, which was later revised and approved by JMM and LY.

## Conflict of Interest

The authors declare that the research was conducted in the absence of any commercial or financial relationships that could be construed as a potential conflict of interest.
